# Kounis Syndrome and Multiorgan Failure Following Multiple Wasp Stings

**DOI:** 10.7759/cureus.14606

**Published:** 2021-04-21

**Authors:** Piratheepan Navaradnam, Navaneethakrishnan Suganthan, Thirunavukarasu Kumanan, Vathulan Sujanitha, Uruthirapasupathi Mayorathan

**Affiliations:** 1 Faculty of Medicine, Teaching Hospital Jaffna, Jaffna, LKA; 2 Medicine, University of Jaffna, Jaffna, LKA; 3 University Medical Unit, Teaching Hospital Jaffna, Jaffna, LKA; 4 Forensic Medicine, Teaching Hospital Jaffna, Jaffna, LKA

**Keywords:** anaphylaxis, myocardial injury, renal failure, rhabdomyolysis, hepatitis, wasp sting, kounis syndrome, disseminated intravascular coagulation

## Abstract

Wasp and bee stings are common in Sri Lanka, and systemic envenomation causes a spectrum of clinical manifestations that includes simple local allergic reaction to life-threatening multiple organ injury or failure. However, wasp toxin-induced acute myocardial infarction is very rare in the literature. Here, we describe a pregnant lady with mass wasp stings who developed toxin-induced acute myocardial infarction with multiorgan injury. The treating physician should anticipate the complications of massive envenomation following multiple wasp stings.

## Introduction

Bees and wasps are biologically classified under phylum of Arthropoda and order of hypmenotrae. It is further divided into three families namely, Apidae (bee), Vespidae (wasp), and Formicidae [1]. Wasp and bee stings are more common in Srilanka [2]. The main pathology of the wasp sting is produced by its venom, which contains a substance that releases histamine, serotonin, thromboxane, prostaglandins and, vasodilating agents [2]. Clinical manifestations of wasp stings vary from a spontaneously resolving localized allergic reaction to multiorgan dysfunction, including allergic angina, known as Kounis syndrome [3]. Herein we describe a 39-year-old previously healthy pregnant woman who developed Kounis syndrome and multiorgan failure following numerous wasp stings.

## Case presentation

A 39-year-old previously healthy female resident of Northern Sri Lanka, with a period of amenorrhea (POA) of 34 weeks and three days (G3P2), presented to the local hospital following numerous wasp stings (>100) all over the body 30 minutes prior. On admission to the local hospital, she had features of allergic reaction with stable clinical parameters including blood pressure (BP) of 150/70 mmHg, heart rate of 92 beats per minute, SpO2 of 96% on room air, and Glasgow Coma Scale (GCS) of 15/15. She was immediately treated with intravenous (IV) hydrocortisone 200 mg and IV chlorpheniramine 10 mg before she was transferred to our hospital for further care. She arrived accident and emergency unit within two hours following stings and complained of a burning sensation all over the body, especially around stings, an episode of loose stool with no blood and mucus, and mild difficulty in breathing.

On physical examination, she was alert with a GCS 15/15 and normal vitals, including a BP of 140/90 mmHg, pulse rate of 96 per minute, and SpO2 of 96% on room air. There was evidence of numerous stings, and in some areas, dead wasps were found. Abdominal examination revealed gravid uterus compatible with her POA, and auscultation over the lungs showed no abnormality. Besides, she was noted to have facial edema and edema of both lips with no tongue swelling. She was given 0.5 ml of 1:1,000 intramuscular adrenaline, and all the vitals were monitored very closely. She was also started with supplementary oxygen and intravenous fluids along with other supportive care.

She was assessed by an ENT team with regards to the assessment of the airway. She underwent fiberoptic laryngoscopic examination, and few wasps were removed from the nasal cavity and nasopharynx. It was noted that the airway was patent and adequate, with no evidence of laryngeal edema. Initial evaluation done by obstetrician confirmed viable single fetus.

After 12 hours of admission, urine output started to decline and became dark in color. She was clinically suspected to have myoglobinuria and hemoglobinuria, complicating acute kidney injury with metabolic acidosis. Subsequently, it was confirmed with very high CPK, evidence of intravascular hemolysis along with positive myoglobinuria and hemoglobinuria (Table 1).

**Table 1:** The biochemical profile of the patient is shown with the clinical progression of the disease. ^+^Mild proteinuria; ^++^moderate proteinuria; ^+++^severe proteinuria. MCV: mean corpuscular volume; HCT: hematocrit; ESR: erythrocyte sedimentation rate; CRP: C-reactive protein; AST: aspartate aminotransferase; ALT: alanine aminotransferase; PT/INR: prothrombin time/international normalized ratio; aPTT: activated partial thromboplastin time; CPK: creatine phosphokinase; HPF: high-power field; POA: period of amenorrhea; GCS: Glasgow Coma Scale.

Biochemical investigations	On admission	12 hours after admission	24 hours after admission	36 hours after admission	48 hours after admission	3^rd^ day morning
Full blood count						
White cell count (4,000-11,000/mm^3^)	14.17	28.75	40..45	37.34	31.3	33.2
Neutrophils (50-70%)	88	95	93.9	92.7	93	93.2
Lymphocytes (20-40%)	7.2	3.8	4.7	4.4	4.9	5
Haemoglobin (12-16 g/dl)	11.9	11	11.3	11.3	7.3	7.7
MCV (80-100 fL)	83	81	80.3	78.8	81	80
Red cell count (400,000-550,00 mm^3 ^)	4.53	4.16	4.26	4.16	2.69	3.3
Platelets (150,000-450,000 mm^3^)	415	266	157	58	24	20
HCT (36-44%)	37.7	33	34.2	32.8	21.8	22.1
Inflammatory markers						
ESR (1^st^ hour)	--	--	--	45	--	--
CRP (0-3.0 mg/L)	--	210	267	--	280	--
Renal functions tests						
Blood urea (2.5-6.4 mmol/L)	3.2	--	--	9.9	9.2	9.7
Serum creatinine (53-88 mmol/L)	47	263	287	182	198	248
Serum electrolytes						
Serum sodium (135-145 mmol/L)	138	142	153	158	--	156
Serum potassium (3.5-5.0 mmol/L)	3.9	3.7	3.9	4.5	4.9	4.8
Serum calcium (2.1-2.5 mmol/L)	--	2.28	--	--	2.24	--
Serum phosphorus (2.6-4.5 mg/dL )	--	--	--	4.5	4.6	--
Liver profile						
Serum AST (0-45 U/L)	--	620	355	--	2608	2842
Serum ALT (0-35 U/L)	24	43	357	577	604	3937
Serum bilirubin (0-17.1 mmol/L)	--	17.1	20	27	24	31
Serum protein (64-83g/L)	--	19	16	--	16	--
Clotting profile						
PT/INR (<1.4)	1.3	1.42	1.92	2.1	--	2.6
APTT (<35)	--	42.8	47	84.1	--	44
Serum CPK (U/L)	--	9284	22649	--	--	--
Urine full report						
Protein (+)	+	++	+++	--	--	+++
Pus cells/HPF	10-15	03-05	12-15	--	--	15-20
Red cells/HPF	15-20	40-50	35-40	--	--	25-30
Active sediment (+)	--	--	+			--
Troponin I (<0-0.15 ng/ml)	--	13.3	14.6	30.32	--	--

**Figure 1:**
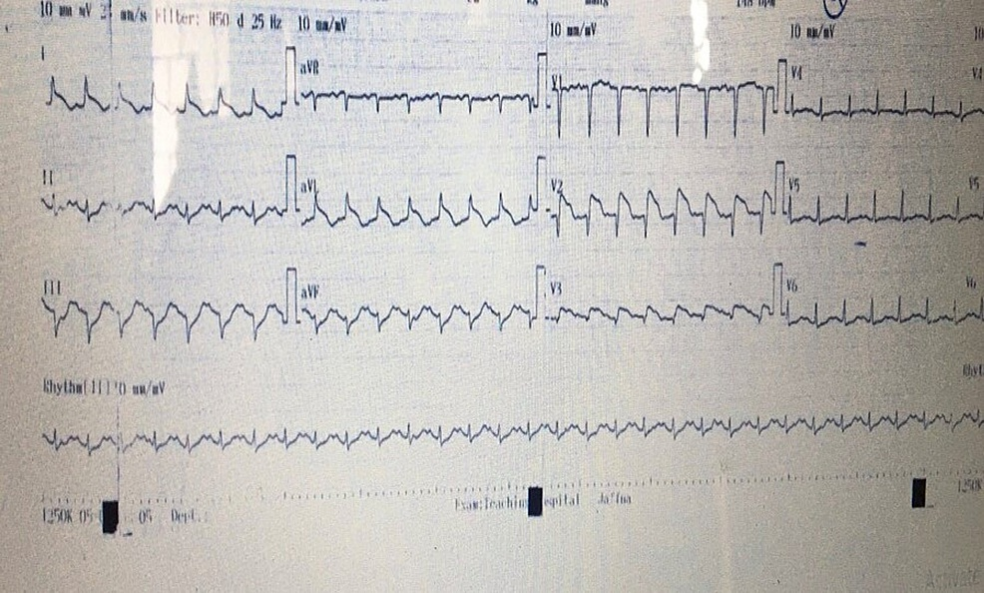
The 12 lead electrocardiography showed ST elevation in leads I, aVL, V1-V3.

She was initiated with continuous renal replacement therapy and infusion of sodium bicarbonate. Further, ultrasound scan of the abdomen showed swollen kidneys compatible with the diagnosis of acute kidney injury. Table 1 summarises the result of the investigations done during the period. While on treatment, she expressed chest discomfort and shortness of breath. ECG taken subsequently showed ST elevation in L1, aVL, and V1-V3 (Figure 1).

Further cardiac assessment showed elevated troponin I and anterior wall hypokinesia with a reduced left ventricular ejection fraction of 35%. She was clinically diagnosed with Kounis syndrome. In the meantime, the ENT team did reevaluation showed moderated laryngeal edema with mild to moderate airway narrowing; she was electively intubated with a difficulty. She also was initiated with a broad-spectrum antibiotic.

After 24 hours of admission, her blood pressure started to drop, and she was managed with IV fluids along with dobutamine and noradrenaline via the central line. However, 12 hours later, she developed ventricular tachycardia, and it was reverted to sinus rhythm following an infusion of amiodarone. As she became hemodynamically unstable in the following hours with dropping BP, adrenaline, and vasopressin were added to maintain the MAP of >65 mmHg. Simultaneously, laboratory evaluation (Table 1) showed evidence of DIC and bedside 2D-echo revealed global hypokinesia with an ejection fraction of 25%-30 %. DIC was managed with blood and blood product. Despite all supportive care, her clinical condition had been deteriorating for the next 12 hours before she went into cardiac arrest following an episode of bradycardia. She succumbed to her illness despite standard resuscitation.

Autopsy findings revealed areas of severe inflamed round patches all over the body and some of which had wasp sting insitu at the center. Generalized edema and laryngeal mucosal edema were noted. The liver was congested and friable. Kidneys were swollen, and cortico medullary pattern was altered. There were no macroscopic changes noted in the heart, and all coronary arteries were free of significant atherosclerotic changes with no occlusive lesions. Lungs showed pulmonary hemorrhage with features of pulmonary edema. The necropsy of the baby didn’t reveal any significant findings.

**Figure 2:**
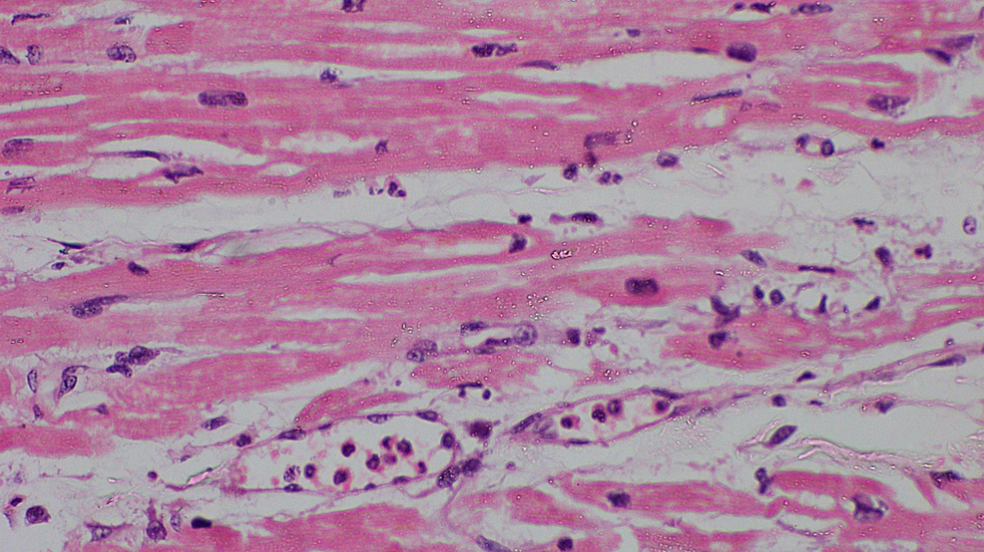
The myocardium shows dilated capillaries containing inflammatory cells with neutrophilextravation. There is an interstitial infiltrate of neutrophils (H&E, X 400). H&E: haematoxylin and eosin.

Histopathology finding of the heart showed myocardial stromal edema, dilated blood vessels, and marginated neutrophils seen in some of those vessels. Wavy fibers and contraction bands were not seen. Occasional faded myocardial nuclei were noted, and there were no chronic changes or fibrosis. All these features were suggestive of early changes of myocardial infarction (Figure 2).

**Figure 3:**
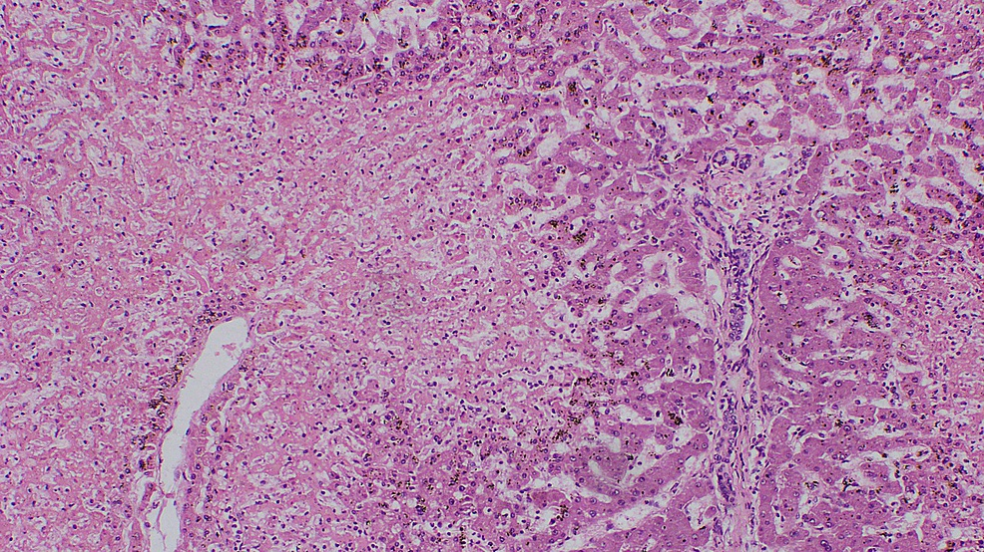
The liver parenchyma showing centrilobular necrosis (H&E, X 100). H&E: haematoxylin and eosin.

**Figure 4:**
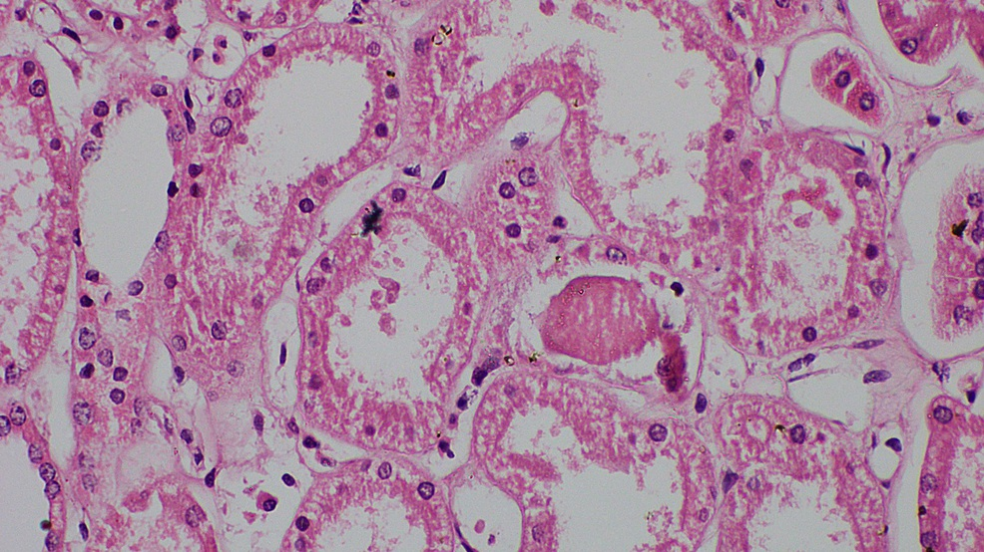
Proximal tubules shows loss of brush border and karyorrhexis (H&E, X 400). H&E: haematoxylin and eosin.

Centrilobular necrosis observed in the liver (Figure 3) and kidney showed evidence of acute tubular necrosis (Figure 4).

 

**Figure 5:**
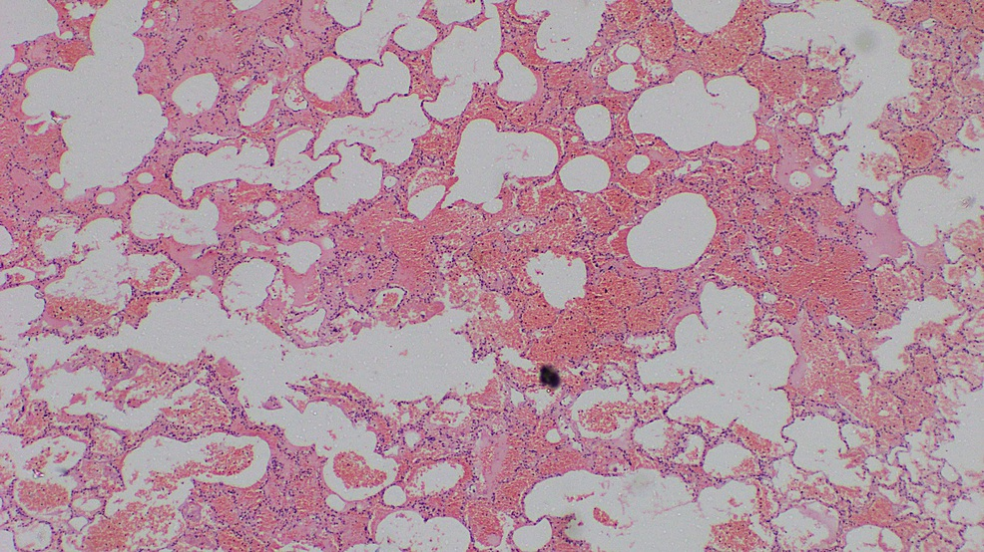
The lung parenchyma shows alveolar haemorrhage and destruction of the alveolar lining epithelium (H&E, X 100). H&E: haematoxylin and eosin.

 Autopsy of the lungs showed alveolar hemorrhage and alveolar destruction (Figure 5).

## Discussion

Patients with a history of numerous wasp stings may have an increased incidence of mortality [4]. Wasps venom-induced organ injury is mainly explained by two mechanisms which include toxin-induced allergic response mainly IgE mediated type 1 hypersensitive reaction and direct effect of toxin causing organ injury, especially in a mass wasp sting. Rhabdomyolysis, hemolysis, DIC, hepatitis, acute kidney injury, myocardial infarction, and pancreatitis are mainly caused by direct toxin-induced injury to the organs [5].

Kounis syndrome is defined as myocardial ischemia or infarction that occurs in the setting of anaphylaxis, and it was first described by Kounis et al. in 1991 [1]. The fundamental mechanism is a venom-induced allergic reaction causing severe coronary vasospasm. Activation of mast cells due to the venom will release inflammatory mediators such as cytokines, leukotrienes, histamine, and serotonin which mediate severe vasoconstriction. Histamine further increases the endogenous release of adrenaline and noradrenaline which will increase myocardial metabolic oxygen consumption thus worsening myocardial ischemia [1].

There are three types of Kounis syndrome explained in the literature [2,6]. In Type 1, toxin induces acute coronary syndrome due to vasoconstriction in patients with previous normal coronary arteries with no risk factors whereas, in Type 2, toxin induces acute coronary syndrome in a patient with preexisting atherovascular disease. Type 3 is caused by thrombus formation in the stent due to drug-eluting stent causing hypersensitive reaction [2,6]. In our patient, ECG showed evidence of ST-elevation myocardial infarction involving leads L1, aVL, and V1-V3 along with raising cardiac troponin. 2D-ECHO done subsequently showed anterior wall hypokinesia with reduced ejection fraction. An autopsy examination of the heart revealed a normal coronary artery with no macroscopic evidence of infarction and histology confirmed the early infarction (Figure 2). Based on the above finding, a diagnosis of Kounis syndrome Type 1 was made. Management of Kounis syndrome has been a challenge for treating physicians. Type 1 can be managed with hydrocortisone and chlorpheniramine alone [6]. In addition, Calcium channel blockers and nitrates can be used to relieve vasospasm provided normal blood pressure. Conventional antiplatelets with anticoagulation can be used in Type 2 Kounis syndrome along with corticosteroids and antihistamine [6]. Management of Type 3 includes mast cell stabilizer, steroid, and antihistamine [6]. Patient with anterior wall involvement has the worst outcome than with inferior wall involvement [6]. A pathological autopsy will be helpful to establish the cause of death and further identification of toxin-mediated injury [6]

Multiple wasp stings are known to cause injury to other organs such as lungs, kidneys, and liver in addition to the heart [1]. A direct effect of the toxin causes lung injury. Our patient’s autopsy demonstrates pulmonary hemorrhage with pulmonary edema, and histology revealed focal alveolar destruction (Figure 3). In addition, liver injury was also caused by persistent systemic hypotension causing ischemic hepatitis [5]. In our patient macroscopic autopsy showed fatty changes, and liver histology revealed centrilobular necrosis (Figure 4).

There are several mechanisms which contribute to acute kidney injury in multiple wasp bite. These include acute tubular necrosis, interstitial nephritis due to a direct effect of the toxin, and pigmented nephropathy secondary to intravascular hemolysis and rhabdomyolysis, hypoperfusion causing pre-renal type renal failure due to shock [4]. In our patient, histology of the kidney showed acute tubular necrosis (Figure 5). As the outcome of the patient depends on the rapid recovery of acute kidney injury, therefore early management of acute kidney injury in a patient with wasp sting is crucial [4]. Initial treatment options include optimal hydration, alkalization of urine, which reduces the need for renal replacement therapy [4]. If needed, early renal replacement therapy such as continuous renal replacement therapy and intermittent hemodialysis should be provided because acidosis will worsen the recovery of organ injury [4].

## Conclusions

This case illustrates severe wasp sting which causes multiorgan dysfunction, including rare complications of Kounis syndrome. The treating physician should anticipate the occurrence of even rare complications of multiple wasp stings such as Kounis syndrome, myocarditis, rhabdomyolysis, and disseminated intravascular coagulation. Treatment and better outcomes remain challenging in massive poisoning following multiple wasp stings.
